# Spiritual and Religious Influences on Healthcare Professionals’ Career Meaning and Perceptions of Aging

**DOI:** 10.1093/geroni/igaf122.4012

**Published:** 2025-12-31

**Authors:** Heidi Ewen, Anne Fish, James Fish, Asiya Patel

**Affiliations:** SUNY Binghamton University, Binghamton, New York, United States; SUNY Binghamton University, Binghamton, New York, United States; SUNY Binghamton University, Binghamton, New York, United States; SUNY Binghamton University, Binghamton, New York, United States

## Abstract

Religious and spiritual beliefs play a significant role in shaping healthcare professionals’ attitudes, behaviors, and perceptions of patient care. This study explores how personal spirituality and religiosity contribute to professionals’ sense of meaning in their careers and influence their views on aging. Participants reported their levels of spirituality and religiosity using a four-point scale and completed two reliable instruments: the Spiritual Meaning of Healthcare Career scale (α = .84) and the Aging as Punishment for Sin subscale (α = .88). ANOVA results revealed that those identifying as “very” or “moderately” religious or spiritual scored significantly higher on the Spiritual Meaning scale. While most participants disagreed with the notion that aging is a punishment for sin, individuals who were “very religious” scored higher on this belief subscale. Regression analyses revealed that spiritual intensity was a strong predictor of the perception of aging as punishment for sin, while both spiritual and religious intensity were significant predictors of the spiritual meaning of a healthcare career. Notably, the Aging as Punishment for Sin subscale demonstrated strong psychometric validity and holds promise for future research, particularly in understanding and addressing ageist beliefs in clinical settings. Interestingly, even among those endorsing this belief, highly religious participants expressed less overall negativity toward aging. These findings underscore the complex interplay between faith and professional identity and highlight the need for nuanced consideration of spirituality in healthcare ethics and education.

